# Rare variant association analysis in case-parents studies by allowing for missing parental genotypes

**DOI:** 10.1186/s12863-018-0597-8

**Published:** 2018-01-15

**Authors:** Yumei Li, Yang Xiang, Chao Xu, Hui Shen, Hongwen Deng

**Affiliations:** 1 School of Mathematics and Computational Science, Huaihua University, Huaihua, Hunan 418008 People’s Republic of China; 2 Center for Bioinformatics and Genomics, Department of Global Biostatistics and Data Science, Tulane University, New Orleans, LA 70112 USA; 3 Center for Bioinformatics and Genomics, School of Public Health and Tropical Medicine, Tulane University, New Orleans, LA 70112 USA

**Keywords:** Rare-variant association analysis, Case-parent trios, Collapsing method

## Abstract

**Background:**

The development of next-generation sequencing technologies has facilitated the identification of rare variants. Family-based design is commonly used to effectively control for population admixture and substructure, which is more prominent for rare variants. Case-parents studies, as typical strategies in family-based design, are widely used in rare variant-disease association analysis. Current methods in case-parents studies are based on complete case-parents data; however, parental genotypes may be missing in case-parents trios, and removing these data may lead to a loss in statistical power. The present study focuses on testing for rare variant-disease association in case-parents study by allowing for missing parental genotypes.

**Results:**

In this report, we extended the collapsing method for rare variant association analysis in case-parents studies to allow for missing parental genotypes, and investigated the performance of two methods by using the difference of genotypes between affected offspring and their corresponding “complements” in case-parent trios and TDT framework. Using simulations, we showed that, compared with the methods just only using complete case-parents data, the proposed strategy allowing for missing parental genotypes, or even adding unrelated affected individuals, can greatly improve the statistical power and meanwhile is not affected by population stratification.

**Conclusions:**

We conclude that adding case-parents data with missing parental genotypes to complete case-parents data set can greatly improve the power of our strategy for rare variant-disease association.

**Electronic supplementary material:**

The online version of this article (10.1186/s12863-018-0597-8) contains supplementary material, which is available to authorized users.

## Background

The development of next-generation sequencing technologies has facilitated association studies of rare variants (minor allele frequency (MAF) < 1%). Family-based design, as an important strategy in genetic studies (especially for rare variants) for human complex diseases, has some advantages over population-based design [1, 2]. The most prominent advantage is that many family-based association methods can effectively control for population admixture and substructure which is more prominent for rare variants and thus avoid spurious associations due to population admixture or substructure [3, 4]. Moreover, family-based design can be used to study complex mechanisms, such as parent-of-origin effects and maternally mediated genetic effects, which are difficult to detect with unrelated individuals in population-based design [5]. Case-parents study, as a typical strategy in family-based design, is widely used in rare variant-disease association analysis. For example, combined multivariate and collapsing (CMC) [6], weighted sum statistic (WSS) [7, 8], variable threshold (VT) [9], and the burden of rare variants (BRV) have all been extended into the transmission/disequilibrium test (TDT) [10] framework [11]. Another commonly used method in case-parents study is to treat nontransmitted genotypes of parents to affected offspring as control (also called pseudocontrols or complements) of affected offspring [5, 12, 13]. For example, investigators can construct a difference vector by comparing the genotypes of affected offspring with their corresponding “complements” and use the collapsing method [6, 7] to detect rare causal variants.

A problem with the use of case-parents study is that not all of the parental genotypes (one or both) are available in practice. For example, parents may have died, especially for older patients with Late-Onset diseases such as Alzheimer disease and hypertension, or parents may decline to participate in clinical research. It is often difficult to recruit large enough samples for case-parents study, especially for rare disease, and thus the sample size is generally small. Discarding those families with missing one or both parental genotypes can lead to statistical power loss. Statistical methods in case-parents study allowing for missing parental genotypes have been widely developed for common variant-disease association analysis [14, 15]. However, few works discuss rare variant-disease association in case-parents study when parental genotypes are missing. Because missing both parental genotypes implies case only (or unrelated affected individuals), allowing for missing one parental genotypes or case only will increase sample size in case-parents study and thus may enhance statistical power for rare variant association analysis. Therefore, it is useful to develop statistical approaches in case-parents study by allowing for missing parental genotypes to test rare variant-disease association.

In this report, we will extend the collapsing method for rare variant association analysis to case-parents study by using the genotype difference of affected offspring with their corresponding “complements” in case-parents trios and TDT framework. Our strategy allows for missing one or both parental genotypes (or case only). We develop our strategy in homogenous populations. Through simulation studies, we investigate the performance of the proposed method in a homogenous population as well as in populations with population stratification under three scenarios: complete case-parents data mixed with one parental genotypes missing, complete case-parents data mixed with both parental genotypes missing, and complete case-parents data mixed with one and both parental genotypes missing.

## Methods

In this study, all datasets were publically available and no research requiring ethics approval was conducted.

### Notation

Consider a data set in a homogenous population Ω = {Ω_**0**_, Ω_**Ι**_, Ω_**ΙΙ**_} consists of three types of case-parents trios with the genotype of affected offspring known in each family. Ω_**0**_, Ω_**Ι**_, and Ω_**ΙΙ**_ denote three types of case-parents trios when there are 0, 1, and 2 missing parental genotypes, respectively. We consider three combinations of Ω_**0**_, Ω_**Ι**_, and Ω_**ΙΙ**_: Ω_**0** + **Ι**_ = {Ω_**0**_, Ω_**Ι**_}, Ω_**0** + **ΙΙ**_ = {Ω_**0**_, Ω_**ΙΙ**_}, and Ω_**0**+**Ι**+**ΙΙ**_ = {Ω_**0**_, Ω_**Ι**_, Ω_**ΙΙ**_}. Ω_**0** + **Ι**_ is the samples data set consisting of complete case-parents trio with the known genotypes for each member in the trio (Ω_**0**_) and case-parents trio with missing one parental genotype (type Ω_**Ι**_). Ω_**0** + **ΙΙ**_ is the samples data set consisting of type Ω_**0**_ and type Ω_**ΙΙ**_ with missing genotypes of both parents. Ω_**0**+**Ι**+**ΙΙ**_ includes sample data of all types of Ω_**0**_, Ω_**Ι**_, and Ω_**ΙΙ**_. We assume N case-parents trios with N_0_, N_I_, N_II_ trios for Ω_**0**_, Ω_**Ι**_, and Ω_**ΙΙ**_, respectively, are sampled (N = N_0_+ N_I_ + N_II_). Let *G*_*O*_ be the minor allele count carried by the affected offspring. Let {*G*_F_, *G*_M_} be the minor allele count carried by parents in a case-parents trio. The curly braces indicate set notation rather than ordered pairs. For example, {*G*_F_, *G*_M_} = {1, 2} means *G*_F_ = 1, *G*_M_ = 2 or *G*_F_ = 2, *G*_M_ = 1. Let a triplet ({*G*_*F*_, *G*_*M*_}, *G*_*O*_) be a case-parents trio.

### Rare variants association analysis

Let *x* = 2*G*_*O*_ − *G*_*F*_ − *G*_*M*_ be the paired difference in genotypes between the affected offspring and the complement (pseudo-control). We consider *k* variants with *q* causal variants in an interesting region, e.g., a gene region. The variants and case-parents trios are indexed by *i* and *j* (*i* = 1, ⋯, *k*; *j* = 1, 2, ⋯, *N*), respectively. We redefine a paired difference ${\tilde{x}}_{ij}$ for *j*th trio at *i*th variant as flowing,

1 $$ {\tilde{x}}_{ij}=\left\{\begin{array}{c}{x}_{ij},\kern14em \left(\left\{{G}_{Fij},{G}_{Mij}\right\},{G}_{Oij}\right)\in {\Omega}_0\\ {}E\left({x}_{ij}|{G}_{Oij}|{G}_{Fij}\right)\ \mathrm{or}\ E\left({x}_{ij}|{G}_{Oij}|{G}_{Mij}\right),\left(\left\{{G}_{Fij},{G}_{Mij}\right\},{G}_{Oij}\right)\in {\Omega}_{\mathbf{I}}\\ {}E\left({x}_{ij}|{G}_{Oij}\right),\kern11.2em \left(\left\{{G}_{Fij},{G}_{Mij}\right\},{G}_{Oij}\right)\in {\Omega}_{\mathbf{I}\mathbf{I}}\end{array}\right. $$

We can calculate *E*(*x*|⋅) under the assumption of random mating (thus Hardy-Weinberg equilibrium) and with the rule of genetic inheritance if ({*G*_*F*_ , *G*_*M*_}, *G*_*O*_) ∈ Ω_**Ι**_ *or* Ω_**ΙΙ**_. For example, when ({*G*_*F*_ , *G*_*M*_}, *G*_*O*_) ∈ Ω_**ΙΙ**_, *P*{{*G*_*F*_ , *G*_*M*_} = {1, 1}| *G*_*O*_ = 2} = (1 − MAF)^2^, *P*{{*G*_*F*_ , *G*_*M*_} = {1, 2}| *G*_*O*_ = 2} = 2MAF ⋅ (1 − MAF), and *P*{{*G*_*F*_ , *G*_*M*_} = {2, 2}| *G*_*O*_ = 2} = MAF^2^, then *P*{*x* = 0| *G*_*O*_ = 2} = *P*{{*G*_*F*_ , *G*_*M*_} = {2, 2}| *G*_*O*_ = 2} = MAF^2^, *P*{*x* = 1| *G*_*O*_ = 2} = *P*{{*G*_*F*_ , *G*_*M*_} = {1, 2}| *G*_*O*_ = 2} = 2MAF ⋅ (1 − MAF), and *P*{*x* = 2| *G*_*O*_ = 2} = *P*{{*G*_*F*_ , *G*_*M*_} = {1, 1}| *G*_*O*_ = 2} = (1 − MAF)^2^. Thus

2 $$ E\left(x|{G}_O=2\right)=\underset{i=0}{\overset{2}{\Sigma}}P\left\{x=i|{G}_O=2\right\}\cdot i=2\left(1-\mathrm{MAF}\right) $$

We use the known parental genotypes or the background-population of samples to estimate MAF. Other *E*(*x*| ⋅) can be calculated similar to Eq. (2).

The collapsing method for rare variants can be directly extended to family-based study with the difference vectors in case-parents data. We denote this method as *Z*_*c*_ which can be defined as

3 $$ {Z}_C=\frac{U}{\sqrt{Var(U)}} $$

where $U={\mathbf{1}}^T\overline{X}$, **1** is a *k*-dimensional vector **1** = (1, ⋯, 1)^*T*^, $\overline{X}=\frac{1}{N}{\left(\underset{j=1}{\overset{N}{\Sigma}}{x}_{1j},\underset{j=1}{\overset{N}{\Sigma}}{x}_{2j},\cdots, \underset{j=1}{\overset{N}{\Sigma}}{x}_{kj}\right)}^T$, ${\sigma}_{ij}=\frac{1}{\left(N-1\right)}\underset{r,\mathrm{s}=1}{\overset{N}{\Sigma}}\left({x}_{ir}-\frac{1}{N}\underset{r=1}{\overset{N}{\Sigma}}{x}_{ir}\right)\left({x}_{js}-\frac{1}{N}\underset{s=1}{\overset{N}{\Sigma}}{x}_{js}\right),$ and $Var(U)=\frac{1}{N}\underset{\mathrm{i},\mathrm{j}=1}{\overset{k}{\Sigma}}{\sigma}_{ij}$. When consider missing parental genotypes, we substitute ${\tilde{x}}_{ij}$ for *x*_*ij*_ and denote the test statistic by ${\tilde{Z}}_C$,

4 $$ {\tilde{Z}}_C=\frac{\tilde{U}}{\sqrt{Var\left(\tilde{U}\right)}} $$

In the TDT framework, we let *b*_*ij*_ be the number of the minor allele transmitted from heterozygous parent to the affected offspring at variant *i* in *j*th trio and *c*_*ij*_ be the number of the major allele transmitted from heterozygous parent to the affected offspring at variant *i* in *j*th trio. Let ${b}_i=\underset{j}{\Sigma}{b}_{ij}$be the total number of minor-allele-transmitted from heterozygous parents to the affected offspring at *i*th variant and ${c}_i=\underset{j}{\Sigma}{c}_{ij}$ is the total number of major-allele-transmitted from heterozygous parents to the affected offspring at variant *i*. The collapsing method for rare variants in TDT framework (corresponding to TDT_BRV_ in He et al. 2014) is

5 $$ {TDT}_{\mathrm{BRV}}=\frac{{\left({\Sigma}_i^k{b}_i-{\Sigma}_i^k{c}_i\right)}^2}{\Sigma_i^k{b}_i+{\Sigma}_i^k{c}_i} $$

When consider missing parental genotypes, we define ${\tilde{c}}_{ij}$ and ${\tilde{b}}_{ij}$ as following,

$$ {\tilde{b}}_{ij}=\left\{\begin{array}{c}\ {b}_{ij},\kern6em \left(\left\{{G}_{Fij},{G}_{Mij}\right\},{G}_{Oij}\right)\in {\Omega}_0\ \\ {}E\left({b}_{ij}|{G}_{Oij},{G}_{Fij}\right),\kern1.4em \left(\left\{{G}_{Fij},{G}_{Mij}\right\},{G}_{Oij}\right)\in {\Omega}_{\mathbf{I}}\\ {}\ E\left({b}_{ij}|{G}_{Oij}\right),\kern3.5em \left(\left\{{G}_{Fij},{G}_{Mij}\right\},{G}_{Oij}\right)\in {\Omega}_{\mathbf{I}\mathbf{I}}\end{array}\right., $$

$$ {\tilde{c}}_{ij}=\left\{\begin{array}{c}{c}_{ij},\kern6em \left(\left\{{G}_{Fij},{G}_{Mij}\right\},{G}_{Oij}\right)\in {\Omega}_0\\ {}E\left({c}_{ij}|{G}_{Oij},{G}_{Fij}\right),\kern0.5em \left(\left\{{G}_{Fij},{G}_{Mij}\right\},{G}_{Oij}\right)\in {\Omega}_{\mathbf{I}}\\ {}E\left({c}_{ij}|{G}_{Oij}\right),\kern2.75em \left(\left\{{G}_{Fij},{G}_{Mij}\right\},{G}_{Oij}\right)\in {\Omega}_{\mathbf{I}\mathbf{I}}\end{array}\right. $$

In Additional file 1: Table S1 and Additional file 2: Table S2 present all the expectations of*E*(*x*| ⋅), *E*(*b*| ⋅), and *E*(*c*| ⋅) when ({*G*_*F*_, *G*_*M*_}, *G*_*O*_) ∈ Ω_**Ι**_ and ({*G*_*F*_, *G*_*M*_}, *G*_*O*_) ∈ Ω_**ΙΙ**_, respectively. We substitute ${\tilde{b}}_{ij}$ and ${\tilde{c}}_{ij}$for *b*_*ij*_ and *c*_*ij*_, and denote the test statistic of the TDT_BRV_ method by ${TDT}_{\mathrm{BRV}}$:

6 $$ {TDT}_{BRV}=\frac{{\left({\Sigma}_i^k{\tilde{c}}_i-{\Sigma}_i^k{\tilde{b}}_i\right)}^2}{\Sigma_i^k{\tilde{c}}_i+{\Sigma}_i^k{\tilde{b}}_i} $$

## Results

### Simulation setting

To assess the performance of our method, we perform a series of simulation studies under a wide range of parameter values. The simulation parameter includes the total number of variants (*k*), the MAF of each variant, the number (*q*) and effect size (measured by the odds ratio (OR)) of causal variants, and the sample size (N) for case-parents trios with the number of case-parents trios for Ω_**0**_(N_0_), Ω_**Ι**_(N_I_), and Ω_**ΙΙ**_(N_II_). We simulate two populations.

In the first population, 1000 case-parents families are generated and the parameters are chosen as follows: *k* = 20; *q* = 0.2 *k*, 0.4 *k*, 0.6 *k*, 0.8 *k*; MAF ∈ (0.001, 0.01) with uniform distribution for each variant. Under the null hypothesis of no association, we set OR = 1 for all the variants. Under the alternative hypothesis of association, we set OR = 1 for non-causal variants. Under the alternative hypothesis, two scenarios are considered for the effects of causal variants. First, the causal variants have the same positive direction but different effects. Here we set OR∈[1.2, 3] in arithmetic progression. Second, the causal variants have opposite effects. Here we set OR∈ [0.2, 0.9] ∪[1.2, 3] with half of causal variants belonging to [0.2, 0.9] and the other causal variants belonging to [1.2, 3] in arithmetic progression.

In the second population, 500 case-parents families and a number of unaffected individuals are generated (here, 500 unaffected individuals are generated and they are used to estimate MAF when samples come from the second population). The parameter settings are similar to those in the first population except that the OR of causal variants under the alternative hypothesis. We let the OR of each causal variant in the second population be 0.1 less than that in the first population.

Once the parameter values are chosen, we first generate parental haplotypes based on a latent variable Z = (*Z*_1_, ⋯, *Z*_*k*_) from a multivariate normal distribution with marginal standard normal and covariance structure as described below [16, 17]: if variants *i* and *j* are both causal or both non-causal, then the correlation is set to be Corr (Z_*i*_, Z_*j*_) =0.4^∣*i* − *j*∣^; otherwise the correlation is zero. We transform Z_*i*_ to 0 (major allele) or 1 (minor allele) according to the MAF of the *i*th variant and combine two haplotypes to obtain the parental haplotypes [16, 17]. Offspring haplotypes are generated from the parental haplotype assuming no recombination between the variants. The disease status for an offspring’s phenotype is determined by the following logistic model [18]:

$$ P\left( Affected|{G}_{Oij},i=1,\cdots, k\right)=\frac{1}{1+\exp \left(-\gamma \right)}, $$

$$ \gamma =\ln \left(\frac{c}{1-c}\right)+\underset{i=1}{\overset{k}{\Sigma}}\ln \left(\mathrm{O}{\mathrm{R}}_i\right)\cdot {G}_{Oij} $$

where OR_*i*_ is the odds ratio of *i*th variant, *G*_*Oij*_ is the minor allele count carried by the affected offspring in the *j*th trio at the *i*th variant, and c is the background prevalence of being affected for a subject with no minor alleles. Here, we let c = 0.01 in the first population and c = 0.008 in the second population.

The 1000 case-parents trios in the first population are composed of three types of trios: 500 (N_0_) forΩ_**0**_, 250 for Ω_**Ι**_ by randomly discarding one set of parental genotypes, and 250 for Ω_**ΙΙ**_ by discarding both parental genotypes. There are two types of trios in the 500 case-parents trios in the second population: 250 for Ω_**Ι**_ and 250 for Ω_**ΙΙ**_. In our analysis, we fix N_0_ (=500) and change N_I_ and N_II_. We let N_I_ and N_II_ take the value of $\frac{1}{10}$N_0_, $\frac{1}{5}$N_0_, and $\frac{1}{2}$N_0_. We calculate Z_c_ and *TDT*_BRV_ in Ω_**0**_ and ${\tilde{Z}}_C$ and ${TDT}_{\mathrm{BRV}}$ in Ω_**0** + **Ι**_,Ω_**0** + **ΙΙ**_, and Ω_**0**+**Ι**+**ΙΙ**_. The *p*-value of statistical tests is estimated by a permutation procedure as follows: First calculate the data-based statistic, then recalculate permutation-based statistic by randomly changing signs (positive or negative) of *x*_*ij*_ for ${\tilde{Z}}_C$ and permuting the “transmitted” and “not transmitted” labels randomly for ${TDT}_{\mathrm{BRV}}$ with equal probability. We repeat this process 1000 times and *p*-value is estimated as the proportion of permutation-based statistics that are larger than the data-based statistic. For a given significance level α, the power/type I error rate is estimated as the proportion of rejecting the null hypothesis when *p*-value ≤α with 1000 replicates.

### Type I error rates and power

We investigate the performance of our method in a homogeneous population and in populations with population stratification. For the homogeneous population, all samples come from the first population. For the population stratification, case-parents trios with missing parental genotypes come from the second population. We present in Table 1 the type I error rates when α = 0.05, 0.001. As shown in Table 1, for three situations of Ω_**0** + **Ι**_, Ω_**0** + **ΙΙ**_, and Ω_**0**+**Ι**+**ΙΙ**_, the type I error rates are well-controlled around the nominal levels. This indicates the validity of the method when considering missing one parental genotypes or case only even in population stratification.

**Table 1:** Type I error rate of ${\tilde{Z}}_C$ and ${TDT}_{\mathrm{BRV}}$

	${\tilde{Z}}_C$						${TDT}_{\mathrm{BRV}}$					
	*α* = 0.05			*α* = 0.001			*α* = 0.05			*α* = 0.001		
	Sample size (N)			Sample size (N)			Sample size (N)			Sample size (N)		
Homogeneous population	N_0_ + $\frac{1}{10}$ N_0_	N_0_ + $\frac{1}{5}$ N_0_	N_0_ + $\frac{1}{2}$ N_0_	N_0_ + $\frac{1}{10}$ N_0_	N_0_ + $\frac{1}{5}$ N_0_	N_0_ + $\frac{1}{2}$ N_0_	N_0_ + $\frac{1}{10}$ N_0_	N_0_ + $\frac{1}{5}$ N_0_	N_0_ + $\frac{1}{2}$N_0_	N_0_ + $\frac{1}{10}$ N_0_	N_0_ + $\frac{1}{5}$ N_0_	N_0_ + $\frac{1}{2}$ N_0_
Ω_0 + Ι_	0.057	0.045	0.052	0.0012	0.0014	0.0009	0.053	0.046	0.051	0.0011	0.0012	0.0010
Ω_0 + ΙΙ_	0.055	0.055	0.050	0.0011	0.0008	0.0014	0.054	0.055	0.052	0.0012	0.0009	0.0014
Ω_0 + Ι + ΙΙ_	0.048	0.049	0.046	0.0009	0.0013	0.0012	0.048	0.049	0.049	0.0009	0.0013	0.0013
Population stratification												
Ω_0 + Ι_	0.055	0.051	0.050	0.0013	0.0012	0.0010	0.055	0.047	0.054	0.0013	0.0012	0.0012
Ω_0 + ΙΙ_	0.053	0.056	0.053	0.0014	0.0009	0.0014	0.056	0.054	0.051	0.0012	0.0009	0.0011
Ω_0 + Ι + ΙΙ_	0.049	0.049	0.051	0.0009	0.0013	0.0014	0.048	0.050	0.047	0.0009	0.0010	0.0014

Note: The sample size N = N_0_ + N_I_, N = N_0_ + N_II_, N = N_0_ + N_I_ + N_II_ for Ω_**0** + **Ι**_, Ω_**0** + **ΙΙ**_, and Ω_**0**+**Ι**+**ΙΙ**_, respectively, with N_0_ (=500) complete case-parents trios (Ω_0_). There are N_I_ case-parents trios of Ω_I_ forΩ_**0** + **Ι**_and N_II_ case-parents trios of Ω_II_ for Ω_**0** + **ΙΙ**_ with N_I_, N_II_=$\frac{1}{10}$N_0_, $\frac{1}{5}$N_0_, and $\frac{1}{2}$N_0_, respectively. For Ω_**0**+**Ι**+**ΙΙ**_, N_I_ = N_II_, and N_I_ + N_II_ = $\frac{1}{10}$N_0_, $\frac{1}{5}$N_0_, and $\frac{1}{2}$N_0_, respectively

We present in Tables 2, 3 and 4 the power of ${\tilde{Z}}_C$ and${TDT}_{\mathrm{BRV}}$ in the homogeneous population for three situations of Ω_**0** + **Ι**_, Ω_**0** + **ΙΙ**_, and Ω_**0**+**Ι**+**ΙΙ**_, respectively, when causal variants have the same positive direction but different effects or causal variants have opposite effects. We can see from Table 1 that, when causal variants have different effects with the same direction and the proportion of non-causal variants is 80% or 60%, adding case-parents trios of Ω_**Ι**_ to complete case-parents data set can increase the power of ${\tilde{Z}}_C$ and ${TDT}_{\mathrm{BRV}}$ for rare variants association analysis. For example, when there are 80% non-causal variants, adding $\frac{1}{10}$N_0_ (50), $\frac{1}{5}$N_0_ (100), and $\frac{1}{2}$N_0_(250) case-parents trios of Ω_**Ι**_ to 500 complete case-parents trios improves the powers of ${\tilde{Z}}_C$and${TDT}_{\mathrm{BRV}}$ from 0.408 and 0.602 to 0.566 and 0.712, to 0.674 and 0.748, and to 0.752 and 0.784, respectively. We observed that, although the power of ${\tilde{Z}}_C$ is lower than that of ${TDT}_{\mathrm{BRV}}$with the use of complete case-parents data, adding $\frac{1}{2}$N_0_ case-parents trios of Ω_**Ι**_ to complete case-parents trios helps ${\tilde{Z}}_C$ achieve similar power as that of ${TDT}_{\mathrm{BRV}}$. We also noted that, when the number of non-causal variants is small (40% or 20%), since the two statistics have high power just by using 500 complete case-parents trios, adding case-parents trios of Ω_**Ι**_ does not help to improve power. As we decrease the sample size to 200, adding case-parents trios of Ω_**Ι**_ can still improve power of ${\tilde{Z}}_C$ and ${TDT}_{\mathrm{BRV}}$(data not shown). When causal variants have opposite effects, we also observed that adding case-parents trios of Ω_**Ι**_ can improve the statistical power.

**Table 2:** Empirical power at the 0.05 significance level for Ω_0 + I_ in the homogenous population

		Causal variants have different effects with the same direction		Causal variants have opposite effects	
Non-causal variants	Sample size (N)^a^	${TDT}_{\mathrm{BRV}}$	${\tilde{Z}}_C$	${TDT}_{\mathrm{BRV}}$	${\tilde{Z}}_C$
80%	N_0_	0.602	0.408	0.226	0.140
	+ $\frac{1}{10}$ N_0_	0.712(18.3%)	0.566(38.7%)	0.272(20.4%)	0.228(62.9%)
	+ $\frac{1}{5}$ N_0_	0.748(24.3%)	0.674(65.2%)	0.290(28.3%)	0.257(83.6%)
	+ $\frac{1}{2}$ N_0_	0.784(30.2%)	0.752(84.3%)	0.312(38.1%)	0.304(117%)
60%	N_0_	0.828	0.776	0.364	0.164
	+ $\frac{1}{10}$ N_0_	0.938(13.3%)	0.922(18.8%)	0.458(25.8%)	0.310(89.0%)
	+ $\frac{1}{5}$ N_0_	0.956(15.5%)	0.946(21.9%)	0.506(39.0%)	0.414(152%)
	+ $\frac{1}{2}$ N_0_	0.980(18.4%)	0.982(26.5%)	0.587(61.3%)	0.556(239%)
40%	N_0_	1.00	0.980	0.264	0.178
	+ $\frac{1}{10}$ N_0_	1.00	1.00	0.284(7.58%)	0.242(34.0%)
	+ $\frac{1}{5}$ N_0_	1.00	1.00	0.308(16.7%)	0.296(66.3%)
	+ $\frac{1}{2}$ N_0_	1.00	1.00	0.360(36.4%)	0.350(96.6%)
20%	N_0_	1.00	1.00	0.278	0.166
	+ $\frac{1}{10}$ N_0_	1.00	1.00	0.294(5.76%)	0.218(31.3%)
	+ $\frac{1}{5}$ N_0_	1.00	1.00	0.315(13.3%)	0.275(65.7%)
	+ $\frac{1}{2}$ N_0_	1.00	1.00	0.376(35.2%)	0.330(98.7%)

Note: ^a^ The sample size N = N_0_ + N_I_, denoted by + $\frac{1}{10}$ N_0_, + $\frac{1}{5}$ N_0_, and + $\frac{1}{2}$ N_0_, where there are N_0_ (=500) complete case-parents trios (Ω_0_) and N_I_ case-parents trios of Ω_I_ with N_I_ = $\frac{1}{10}$ N_0_, $\frac{1}{5}$ N_0_, and $\frac{1}{2}$ N_0_, respectively. Shown in parentheses is the proportion of power improvement

**Table 3:** Empirical power at the 0.05 significance level for Ω_0 + II_ in the homogenous population

		Causal variants have different effects with the same direction		Causal variants have opposite effects	
Non-causal variants	Sample size (N)^a^	${TDT}_{\mathrm{BRV}}$	${\tilde{Z}}_C$	${TDT}_{\mathrm{BRV}}$	${\tilde{Z}}_C$
80%	N_0_	0.602	0.408	0.226	0.140
	+ $\frac{1}{10}$ N_0_	0.702(16.6%)	0.551(35.1%)	0.269(19.0%)	0.218(55.7%)
	+ $\frac{1}{5}$ N_0_	0.732(21.6%)	0.660(61.8%)	0.278(23.0%)	0.241(72.1%)
	+ $\frac{1}{2}$ N_0_	0.770(27.9%)	0.745(82.6%)	0.309(36.7%)	0.300(114%)
60%	N_0_	0.828	0.776	0.364	0.164
	+ $\frac{1}{10}$ N_0_	0.920(9.20%)	0.911(17.4%)	0.454(24.7%)	0.310(89.0%)
	+ $\frac{1}{5}$ N_0_	0.941(13.6%)	0.936(20.6%)	0.482(32.4%)	0.406(147%)
	+ $\frac{1}{2}$ N_0_	0.963(16.3%)	0.960(23.7%)	0.575(58.0%)	0.542(230%)
40%	N_0_	1.00	0.980	0.264	0.178
	+ $\frac{1}{10}$ N_0_	1.00	1.00	0.277(4.92%)	0.231(29.8%)
	+ $\frac{1}{5}$ N_0_	1.00	1.00	0.300(13.6%)	0.286(60.7%)
	+ $\frac{1}{2}$ N_0_	1.00	1.00	0.348(31.8%)	0.339(90.4%)
20%	N_0_	1.00	1.00	0.278	0.166
	+ $\frac{1}{10}$ N_0_	1.00	1.00	0.289(3.96%)	0.204(22.9%)
	+ $\frac{1}{5}$ N_0_	1.00	1.00	0.310(11.5%)	0.262(57.8%)
	+ $\frac{1}{2}$ N_0_	1.00	1.00	0.368(32.4%)	0.320(92.8%)

Note: ^a^The sample size N = N_0_ + N_II_, denoted by +$\frac{1}{10}$ N_0_, + $\frac{1}{5}$ N_0_, and + $\frac{1}{2}$ N_0_, where there are N_0_ (=500) complete case-parents trios (Ω_0_) and N_II_ case-parents trios of Ω_II_ with N_II_ = $\frac{1}{10}$ N_0_, $\frac{1}{5}$ N_0_, and $\frac{1}{2}$ N_0_, respectively. Shown in parentheses is the proportion of power improvement

**Table 4:** Empirical power at the 0.05 significance level for Ω_0 + I + II_ in the homogenous population

		Causal variants have different effects with the same direction		Causal variants have opposite effects	
Non-causal variants	Sample size (N)^a^	${TDT}_{\mathrm{BRV}}$	${\tilde{Z}}_C$	${TDT}_{\mathrm{BRV}}$	${\tilde{Z}}_C$
80%	N_0_	0.602	0.408	0.226	0.140
	+ $\frac{1}{10}$ N_0_	0.709(17.8%)	0.560(37.3%)	0.271(19.9%)	0.224(60.0%)
	+ $\frac{1}{5}$ N_0_	0.740(22.9%)	0.668(63.7%)	0.282(24.8%)	0.250(78.6%)
	+ $\frac{1}{2}$ N_0_	0.778(29.2%)	0.749(83.6%)	0.311(37.6%)	0.304(117%)
60%	N_0_	0.828	0.776	0.364	0.164
	+ $\frac{1}{10}$ N_0_	0.930(12.3%)	0.918(18.3%)	0.456(25.3%)	0.302(84.1%)
	+ $\frac{1}{5}$ N_0_	0.949(14.6%)	0.941(21.3%)	0.490(34.6%)	0.410(150%)
	+ $\frac{1}{2}$ N_0_	0.971(17.3%)	0.972(25.3%)	0.581(59.6%)	0.551(236%)
40%	N_0_	1.00	0.982	0.264	0.178
	+ $\frac{1}{10}$ N_0_	1.00	1.00	0.279(5.7%)	0.230(29.2%)
	+ $\frac{1}{5}$ N_0_	1.00	1.00	0.308(16.7%)	0.290(62.9%)
	+ $\frac{1}{2}$ N_0_	1.00	1.00	0.345(30.7%)	0.340(91.0%)
20%	N_0_	1.00	1.00	0.278	0.166
	+ $\frac{1}{10}$ N_0_	1.00	1.00	0.292(5.04%)	0.210(26.5%)
	+ $\frac{1}{5}$ N_0_	1.00	1.00	0.315(13.3%)	0.260(56.6%)
	+ $\frac{1}{2}$ N_0_	1.00	1.00	0.370(33.1%)	0.321(93.3%)

Note: ^a^The sample size N = N_0_++N_I_ + N_II_, denoted by + $\frac{1}{10}$ N_0_, + $\frac{1}{5}$ N_0_, and + $\frac{1}{2}$ N_0_, where there are N_0_ (=500) complete case-parents trios (Ω_0_) and N_I_ case-parents trios of Ω_I_ and N_II_ case-parents trios of Ω_II_ with N_I_ = N_II_ = $\frac{1}{20}$ N_0_, $\frac{1}{10}$ N_0_, and $\frac{1}{4}$ N_0_, respectively. Shown in parentheses is the proportion of power improvement

In order to further show the magnitude of power improvement of ${\tilde{Z}}_C$ and ${TDT}_{\mathrm{BRV}}$, we present in parentheses in Tables 2, 3 and 4 the proportion of power improved by adding case-parents trios of Ω_**Ι**_, Ω_**ΙΙ**_, and Ω_**Ι**+**ΙΙ**_to complete case-parents data set Ω_**0**_. It can be found from Table 2 that the proportion of power improvement drops with a decrease in the number of non-causal variants, and the proportion of power improvement for ${\tilde{Z}}_C$ is higher than that for ${TDT}_{\mathrm{BRV}}$. When causal variants have opposite effects, we observed that the proportion of power improvement is larger than that when causal variants have the same direction. As the proportion of non-causal variants decreases from 80% to 60%, the proportion of power improvement increases. For example, while the powers of ${TDT}_{\mathrm{BRV}}$ and ${\tilde{Z}}_C$ for 80% non-causal variants have improved by 20.4% to 38.1% and 62.9% to 117% with the number of case-parents trios of Ω_**Ι**_ increasing from $\frac{1}{10}$N_0_ to $\frac{1}{2}$N_0_, respectively, the powers of ${TDT}_{\mathrm{BRV}}$ and ${\tilde{Z}}_C$ for 60% non-causal variants have improved by 25.8 to 61.3% and 89.0 to 239%, respectively. However, the proportion of power improvement drops with a further decrease in the number of non-causal variants. For example, with the number of case-parents trios of Ω_**Ι**_ increasing from $\frac{1}{10}$N_0_to $\frac{1}{2}$N_0_, the proportions of power improvement of ${TDT}_{\mathrm{BRV}}$ change from7.58 to 36.4% for 40% non-causal variants and from 5.76 to 35.2% for 20% non-causal variants, respectively, and the proportions of power improvement of ${\tilde{Z}}_C$ change from 34.0 to 96.6% for 40% non-causal variants and from 31.3 to 98.7% for 20% non-causal variants, respectively. This result indicates that, when causal variants have opposite effects, the proportion of power improvement increases early then decreases later with the increase in the number of non-causal variants. Adding case-parents trios of Ω_**Ι**_ with the medium number of non-causal variants is best for power improvement. For Ω_**0** + **ΙΙ**_and Ω_**0**+**Ι**+**ΙΙ**_, Tables 3 and 4 show similar results as those for Ω_**0** + **Ι**_. In addition, we observed that the proportion of power improvement for Ω_**0** + **Ι**_ is the largest among three situations of Ω_**0** + **Ι**_,Ω_**0** + **ΙΙ**_, and Ω_**0**+**Ι**+**ΙΙ**_.

When there is population stratification, Additional file 3: Figure S1-S4 shows the power of ${\tilde{Z}}_C$ and ${TDT}_{\mathrm{BRV}}$against the sample size for various proportions of non-causal variants under three situations of Ω_**0** + **Ι**_,Ω_**0** + **ΙΙ**_, and Ω_**0**+**Ι**+**ΙΙ**_, respectively. The results are similar to those in the homogeneous population. We also consider a general situation for population stratification: Samples from two populations both consist of case-parents trios of three types, Ω_**0**_, Ω_**Ι**_, and Ω_**ΙΙ**_. The simulation results are similar to those in Additional file 3: Figures S1-S4 (data not shown). These results indicate that, when adding case-parents trios with missing parental genotypes or even case only to complete a case-parents data set, population stratification does not affect the power of these two statistics for rare variant association analysis.

## Discussion

In this report, we considered case-parents data with missing parental genotypes for rare variant association analysis in case-parents studies. Based on the collapsing method with the difference vector and TDT framework, we presented two statistics, ${\tilde{Z}}_C$ and ${TDT}_{\mathrm{BRV}}$, allowing for missing parental genotypes. The key in the proposed approach is to estimate the MAF. Actually, in clinical research, experimental design is usually done for a homogenous population or several specific populations. One can use the known parental genotypes or the background-population of samples to estimate MAF. We investigated the performance of these two statistics in three different situations: complete case-parents data mixed with one parental genotype missing, complete case-parents data mixed with both parental genotypes missing, and complete case-parents data mixed with one and both parental genotypes missing. Through simulation studies, we found that adding case-parents data with missing parental genotypes to complete case-parents data set can greatly improve the power of these two statistics, though the proportion of power improvement varied. Additionally, our strategy is not affected by population stratification.

In most studies of disease associations with rare variants, family- and population-based samples were used separately [6, 7, 11, 19, 20]. Although family-based studies have several advantages over population-based studies in rare variant association analysis, it is often difficult to recruit sufficiently large family-based samples, especially for rare diseases. More often, information about parents is incomplete, and this poses some challenges in analysis. Discarding those families with missing parental genotypes will further reduce the sample size and result in a loss of statistical power. In our strategy, case-parents trios missing one or both parental genotypes are kept in analysis and thus can help to greatly improve statistical power. Furthermore, we can see that missing both parental genotypes corresponds to case only. This means we can use unrelated affected individuals in case-parents studies, which is useful for case-parents studies with small sample size. Although population stratification might exist in these unrelated affected individuals recruited from population-based samples, our strategy is not affected by population stratification. Our simulation results showed that combining unrelated affected individuals with complete case-parents data could increase power by 5 ~ 60% for ${TDT}_{\mathrm{BRV}}$and 20 ~ 200% for${\tilde{Z}}_C$ in both homogenous populations and populations with population stratification.

In addition to allowing for missing parental genotypes, our method can be used to address another problem when there are missing genotypes for individual variants in parental data. In fact, when individual variants are analyzed and there are missing genotypes for some variants, removing those samples for variants with missing genotypes will result in inconsistency of the sample size. With the strategy described above, our method can overcome this problem. However, our strategy is not suitable for case-parents trios with missing offspring genotypes, so further study is needed to address such scenarios.

## Conclusions

The proposed strategy allowing for missing parental genotypes, or even adding unrelated affected individuals, can greatly improve the statistical power for rare variant-disease association and meanwhile is not affected by population stratification.

## Additional files

Additional file 1: Table S1. All the expectations of E(x),E(b),and E(c) when (G_F_, G_M_ ,G_O_)∈Ω_I_. (PDF 109 kb)

Additional file 2: Table S2. All the expectations of E(x),E(b),and E(c) when (G_F_, G_M_ ,G_O_)∈Ω_II_. (PDF 88 kb)

Additional file 3: Figure S1.Empirical power against the sample size at the 0.05 significance level in population stratification when there are 20% non-causal variants. Note: A and B are for${\tilde{Z}}_C$, and C and D are for ${TDT}_{\mathrm{BRV}}$when causal variants have different effects with the same direction and causal variants have opposite effects, respectively. The sample size N=N_0_, N_0_ + 1/10 N_0_, N_0_ + 1/5 N_0_, N_0_ + 1/2 N_0_ with N_0_ = 500 denoted by 0, 1/10, 1/5, and 1/2 respectively. Ω_0 + I_ (○), Ω_0 + II_ (*), Ω_0 + I + II_ (+). **Figure S2.** Empirical power against the sample size at the 0.05 significance level in population stratification when there are 40% non-causal variants. Note: A and B are for${\tilde{Z}}_C$, and C and D are for ${TDT}_{\mathrm{BRV}}$ when causal variants have different effects with the same direction and causal variants have opposite effects, respectively. The sample size N=N_0_, N_0_ + 1/10 N_0_, N_0_ + 1/5 N_0_, N_0_ + 1/2 N_0_ with N_0_ = 500 denoted by 0, 1/10, 1/5, and 1/2 respectively. Ω_0 + I_ (○), Ω_0 + II_ (*), Ω_0 + I + II_ (+). **Figure S3.** Empirical power against the sample size at the 0.05 significance level in population stratification when there are 60% non-causal variants. Note: A and B are for ${\tilde{Z}}_C$, and C and D are for ${TDT}_{\mathrm{BRV}}$when causal variants have different effects with the same direction and causal variants have opposite effects, respectively. The sample size N=N_0_, N_0_ + 1/10 N_0_, N_0_ + 1/5 N_0_, N_0_ + 1/2 N_0_ with N_0_ = 500 denoted by 0, 1/10, 1/5, and 1/2 respectively. Ω_0 + I_ (○), Ω_0 + II_ (*), Ω_0 + I + II_ (+). **Figure S4.** Empirical power against the sample size at the 0.05 significance level in population stratification when there are 80% non-causal variants. Note: A and B are for ${\tilde{Z}}_C$, and C and D are for ${TDT}_{\mathrm{BRV}}$ when causal variants have different effects with the same direction and causal variants have opposite effects, respectively. The sample size N=N_0_, N_0_ + 1/10 N_0_, N_0_ + 1/5 N_0_, N_0_ + 1/2 N_0_ with N_0_ = 500 denoted by 0, 1/10, 1/5, and 1/2 respectively. Ω_0 + I_ (○), Ω_0 + II_ (*), Ω_0 + I + II_ (+). (PDF 89 kb)

## Abbreviations

BRV: Burden of rare variants
CMC: Combined multivariate and collapsing
LD: Linkage disequilibrium
MAF: Minor allele frequency
OR: Odds ratio
TDT: Transmission/disequilibrium test
VT: Variable threshold
WSS: Weighted sum statistic
