# Supplementary material for: Rare variant association analysis in case-parents studies by allowing for missing parental genotypes
Source: BMC Genet. 2018 Jan 15;19:7. doi: 10.1186/s12863-018-0597-8 (PMC5769338; doi:10.1186/s12863-018-0597-8)
Supplement: Supplementary file 1 — All the expectations of E(x),E(b),and E(c) when (GF, GM ,GO)∈ΩI. (PDF 109 kb) [file 12863_2018_597_MOESM1_ESM.pdf]

Table S1. All the expectations of $E(x)$, $E(b)$, and $E(c)$ when $(G_F,\ G_M,\ G_O)\in\Omega_{\mathbf{I}}$.

G_O	G_F	x	$P\{G_M \mid G_F, G_O\}$	b	c	$E(b \mid G_O, G_F)$	$E(c \mid G_O, G_F)$	$E(x \mid G_O, G_F)$
$G_O=0$	$G_F=0$	$x=-1$	$P\{G_M=1 \mid G_F, G_O\}=\text{MAF}$	0	1	0	MAF	$-\text{MAF}$
		$x=0$	$P\{G_M=0 \mid G_F, G_O\}=1-\text{MAF}$	-	-			
	$G_F=1$	$x=-2$	$P\{G_M=1 \mid G_F, G_O\}=\text{MAF}$	0	2	0	$1+\text{MAF}$	$-(1+\text{MAF})$
		$x=-1$	$P\{G_M=0 \mid G_F, G_O\}=1-\text{MAF}$	0	1			
$G_O=1$	$G_F=0$	$x=0$	$P\{G_M=2 \mid G_F, G_O\}=\text{MAF}$	-	-	$1-\text{MAF}$	0	$1-\text{MAF}$
		$x=1$	$P\{G_M=1 \mid G_F, G_O\}=1-\text{MAF}$	1	0			
	$G_F=1$	$x=-1$	$P\{G_M=2 \mid G_F, G_O\}=\text{MAF}^2$	0	1	$1-\text{MAF}^2$	$2\text{MAF}-\text{MAF}^2$	$1-2\text{MAF}$
		$x=0$	$P\{G_M=1 \mid G_F, G_O\}=2\text{MAF}\cdot(1-\text{MAF})$	1	1			
		$x=1$	$P\{G_M=0 \mid G_F, G_O\}=(1-\text{MAF})^2$	1	0			
	$G_F=2$	$x=-1$	$P\{G_M=1 \mid G_F, G_O\}=\text{MAF}$	0	1	0	MAF	$-\text{MAF}$
		$x=0$	$P\{G_M=0 \mid G_F, G_O\}=1-\text{MAF}$	-	-			
$G_O=2$	$G_F=2$	$x=0$	$P\{G_M=2 \mid G_F, G_O\}=\text{MAF}$	-	-	$1-\text{MAF}$	0	$1-\text{MAF}$
		$x=1$	$P\{G_M=1 \mid G_F, G_O\}=1-\text{MAF}$	1	0			
	$G_F=1$	$x=1$	$2P\{G_M=2 \mid G_F, G_O\}=\text{MAF}$	1	0	$2-\text{MAF}$	0	$2-\text{MAF}$
		$x=2$	$2P\{G_M=1 \mid G_F, G_O\}=1-\text{MAF}$	2	0			
