# Supplementary material for: Rare variant association analysis in case-parents studies by allowing for missing parental genotypes
Source: BMC Genet. 2018 Jan 15;19:7. doi: 10.1186/s12863-018-0597-8 (PMC5769338; doi:10.1186/s12863-018-0597-8)
Supplement: Supplementary file 2 — All the expectations of E(x),E(b),and E(c) when (GF, GM ,GO)∈ΩII. (PDF 88 kb) [file 12863_2018_597_MOESM2_ESM.pdf]

Table S2. All the expectations of $E(x)$, $E(b)$, and $E(c)$ when $(G_F,\ G_M,\ G_O) \in \Omega_{\mathbf{II}}$.

G_O	x	$P\{\ G_M, G_F \mid G_O\}$	b	c	$E(b \mid G_O)$	$E(c \mid G_O)$	$E(x \mid G_O)$
$G_O = 0$	$x = -2$	$P\{G_F = 1,\ G_M = 1 \mid G_O\} = \text{MAF}^2$	0	2	0	2MAF	-2MAF
	$x = -1$	$P\{G_F = 0,\ G_M = 1 \mid G_O\} = 2\text{MAF} \cdot (1 - \text{MAF})$	0	1			
	$x = 0$	$P\{G_F = 0,\ G_M = 0 \mid G_O\} = (1 - \text{MAF})^2$	-	-			
$G_O = 1$	$x = -1$	$P\{G_F = 1,\ G_M = 2 \mid G_O\} = \text{MAF}^2$	0	1	$1 - \text{MAF}$	MAF	$1 - 2\text{MAF}$
	$x = 0$	$(G_F = 1,\ G_M = 1) \mid G_O\} = \text{MAF} \cdot (1 - \text{MAF})$	1	1			
		$P\{(G_F = 0,\ G_M = 2) = \text{MAF} \cdot (1 - \text{MAF})$	-	-			
	$x = 1$	$P\{G_F = 0,\ G_M = 1 \mid G_O\} = (1 - \text{MAF})^2$	1	0			
$G_O = 2$	$x = 0$	$P\{G_F = 2,\ G_M = 2 \mid G_O\} = \text{MAF}^2$	-	-	$2(1 - \text{MAF})$	0	$2(1 - \text{MAF})$
	$x = 1$	$P\{G_F = 1,\ G_M = 2 \mid G_O\} = 2\text{MAF} \cdot (1 - \text{MAF})$	1	0			
	$x = 2$	$P\{G_F = 1,\ G_M = 1 \mid G_O\} = (1 - \text{MAF})^2$	2	0			
