# Supplementary material for: Rare variant association analysis in case-parents studies by allowing for missing parental genotypes
Source: BMC Genet. 2018 Jan 15;19:7. doi: 10.1186/s12863-018-0597-8 (PMC5769338; doi:10.1186/s12863-018-0597-8)
Supplement: Supplementary file 3 — Empirical power against the sample size at the 0.05 significance level in population stratification when there are 20% non-causal variants. Note: A and B are for${\tilde{Z}}_C$Z˜C, and C and D are for ${TDT}_{\mathrm{BRV}}$TDTBRVwhen causal variants have different effects with the same direction and causal variants have opposite effects, respectively. The sample size N=N0, N0 + 1/10 N0, N0 + 1/5 N0, N0 + 1/2 N0 with N0 = 500 denoted by 0, 1/10, 1/5, and 1/2 respectively. Ω0 + I (○), Ω0 + II (*), Ω0 + I + II (+). Figure S2. Empirical power against the sample size at the 0.05 significance level in population stratification when there are 40% non-causal variants. Note: A and B are for${\tilde{Z}}_C$Z˜C, and C and D are for ${TDT}_{\mathrm{BRV}}$TDTBRV when causal variants have different effects with the same direction and causal variants have opposite effects, respectively. The sample size N=N0, N0 + 1/10 N0, N0 + 1/5 N0, N0 + 1/2 N0 with N0 = 500 denoted by 0, 1/10, 1/5, and 1/2 respectively. Ω0 + I (○) [file 12863_2018_597_MOESM3_ESM.pdf]

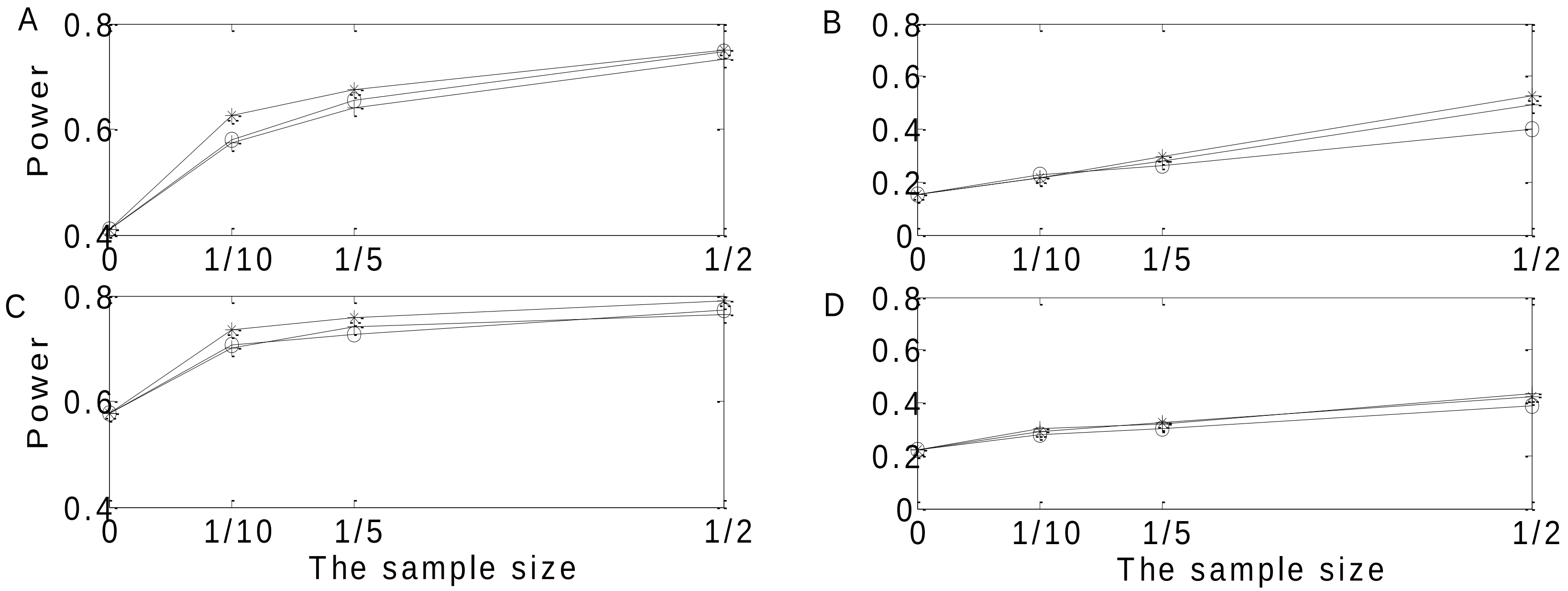

Fig.S1 Empirical power against the sample size at the 0.05 significance level in population stratification when there are 20% non-causal variants.

Note: **A** and **B** are for $\tilde{Z}_C$, and **C** and **D** are for TDT_{BRV} when causal variants have different effects with the same direction and causal variants have opposite effects, respectively. The sample size $N=N_0$, $N_0+1/10N_0$, $N_0+1/5N_0$, $N_0+1/2N_0$ with $N_0=500$ denoted by 0, 1/10, 1/5, and 1/2 respectively. $\Omega_{0+\mathrm{I}}$ (○), $\Omega_{0+\mathrm{II}}$ (*), $\Omega_{0+\mathrm{I+II}}$ (+).

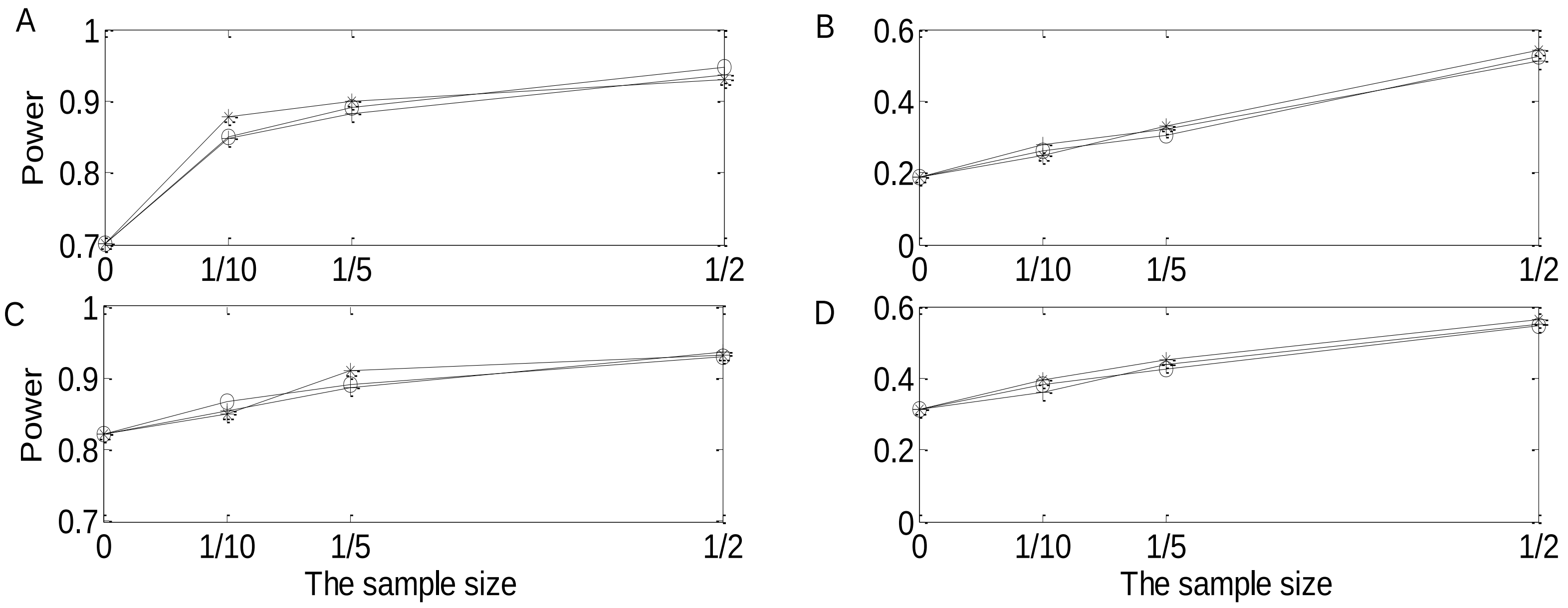

Fig.S2 Empirical power against the sample size at the 0.05 significance level in population stratification when there are 40% non-causal variants.

Note: **A** and **B** are for $\tilde{Z}_C$, and **C** and **D** are for TDT_{BRV} when causal variants have different effects with the same direction and causal variants have opposite effects, respectively. The sample size N=N_0, N_0+1/10N_0, N_0+1/5N_0, N_0+1/2N_0 with N_0=500 denoted by 0, 1/10, 1/5, and 1/2 respectively. $\Omega_{0+\mathrm{I}}$ (○), $\Omega_{0+\mathrm{II}}$ (*), $\Omega_{0+\mathrm{I}+\mathrm{II}}$ (+).

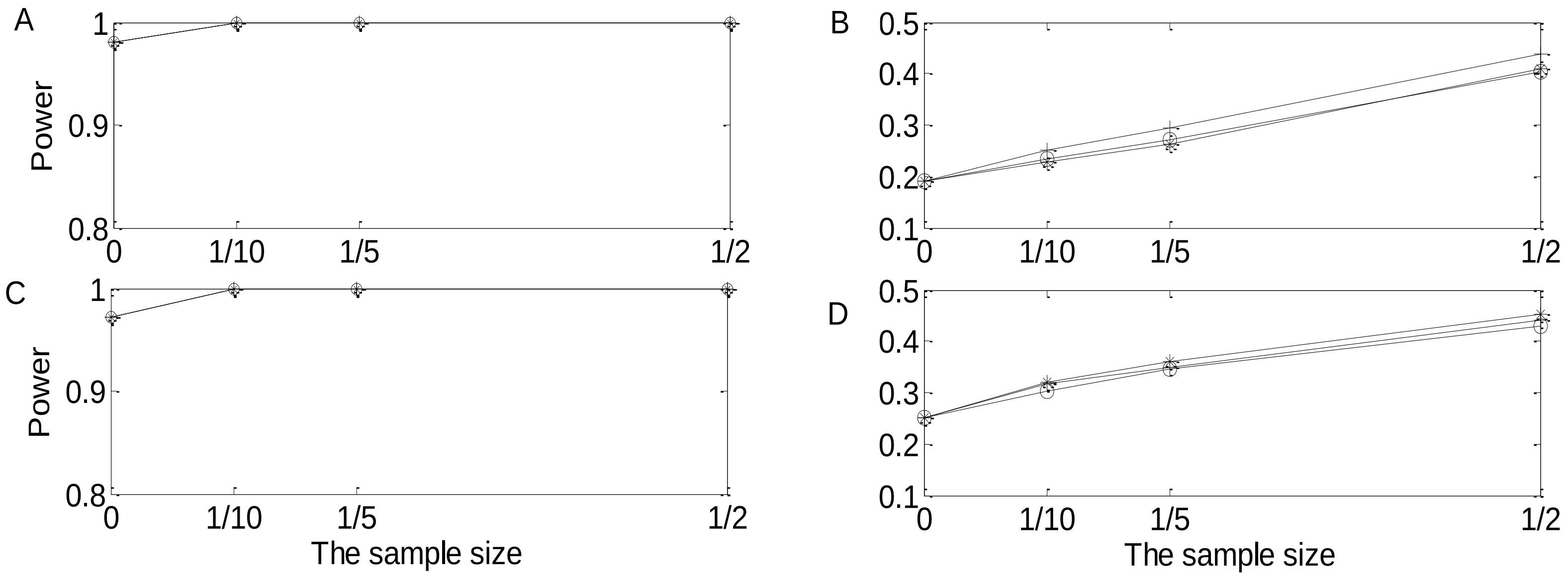

Fig.S3 Empirical power against the sample size at the 0.05 significance level in population stratification when there are 60% non-causal variants.

Note: A and **B** are for $\tilde{Z}_C$, and **C** and **D** are for TDT_{BRV} when causal variants have different effects with the same direction and causal variants have opposite effects, respectively. The sample size $N=N_0$, $N_0+1/10N_0$, $N_0+1/5N_0$, $N_0+1/2N_0$ with $N_0=500$ denoted by 0, 1/10, 1/5, and 1/2 respectively. $\Omega_{0+\mathrm{I}}$ (○), $\Omega_{0+\mathrm{II}}$ (*), $\Omega_{0+\mathrm{I}+\mathrm{II}}$ (+).

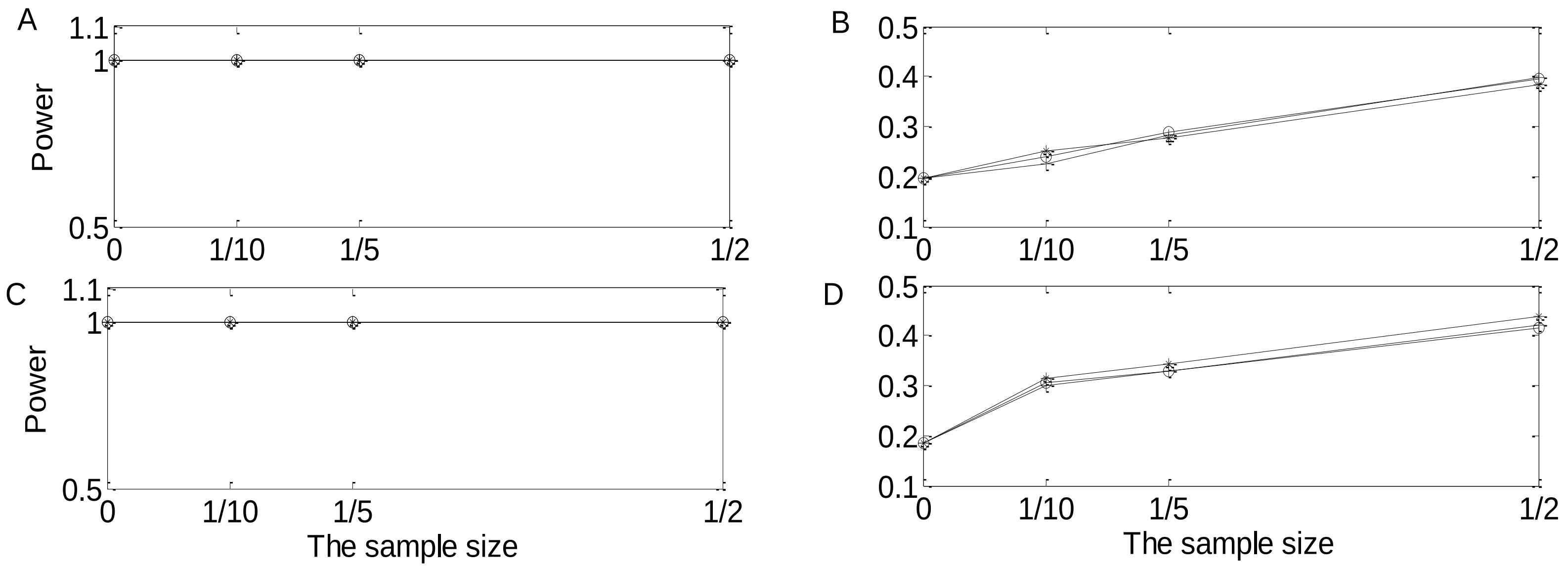

Fig.S4 Empirical power against the sample size at the 0.05 significance level in population stratification when there are 80% non-causal variants.

Note: A and **B** are for $\tilde{Z}_C$, and **C** and **D** are for TDT_{BRV} when causal variants have different effects with the same direction and causal variants have opposite effects, respectively. The sample size $N=N_0$, $N_0+1/10N_0$, $N_0+1/5N_0$, $N_0+1/2N_0$ with $N_0=500$ denoted by 0, 1/10, 1/5, and 1/2 respectively. $\Omega_{0+\mathrm{I}}$ (○), $\Omega_{0+\mathrm{II}}$ (*), $\Omega_{0+\mathrm{I+II}}$ (+).
